# Case of Ossification of the Uterus, Which Was Met with in a Lady Who Died of Strangulated Hernia

**Published:** 1825-12

**Authors:** Thomas Fowkes


ART. VI.—*Case of Ossification of the Uterus, which was met with in a Lady who died of Strangulated Hernia.* By THOMAS FOWKES, Esq.

A LADY, sixty-nine years of age, requested me to see her early on the morning of the 24th of November, 1824. I found her labouring under symptoms of strangulated hernia, which had come on with considerable violence on the preceding evening. She had frequent vomiting, occasional hiccup, and great tenderness of the abdomen, increased on pressure. On examining, I found a hernia on the right side, consisting apparently of a portion of intestine, strangulated under Poupart's ligament, and forming a tumor of the size of a small orange. She had been subject to rupture for about two years, and could not give a clear account of the time when it had last been reduced, as she had never considered it of much consequence.

After bleeding her freely, I endeavoured to return the bowel, but did not succeed: I therefore desired her to send for Mr. Rose, who came there at three in the afternoon. She could not at that time bear pressure on any part of the abdomen; the sickness and hiccup were very distressing; her pulse was quick and sharp, and had risen since she had been bled. Mr. Rose directed the bleeding to be repeated; and, during the faintness produced by it, he succeeded in reducing the hernia, by gentle pressure continued for some time. Fomentations were applied to the abdomen, and a weak solution of Epsom salts given in small and repeated doses. She had one or two watery evacuations an hour or two after the bowel was returned; but the sickness, which had for a time become more moderate, increased towards night, and she died between one and two on the following morning.

Mr. Rose having expressed a particular wish to examine the body, I obtained permission for him to do so. The following is the account which he drew up of the appearances on dissection:—

The portion of intestine which had been strangulated was found to consist of about three inches of the lower part of the ileum. A deep indented line, from ulceration of the peritoneal coat, both at the upper and lower part of this portion, marked the great extent of pressure it had undergone. Between these the gut was of a bluish colour, and was evidently advancing rapidly to gangrene. From the great pressure, and from her advanced age, it had not recovered itself on being returned into the abdomen. The intestines were generally glued together by inflammation of the peritoneum.

The uterus projected into the middle of the cavity of the pelvis, in a pyramidal form, and appeared to consist of an entire mass of bone. On taking it out, for the purpose of more careful examination, and making a section through its anterior portion, which was still fleshy, the triangular cavity was found entire, though much compressed and reduced in extent. A large spherical mass of bone, of the size of a pullet's egg, was imbedded in the uterus, behind the cavity; part of the substance of the uterus being distinctly to be traced over its upper part.

There was a hard rounded tubercle of the uterus imbedded also in its substance, a little below one of the fallopian tubes. This was of a white appearance, of the size of a hazel nut, and, when cut into, was found to exhibit the common structure of these bodies, being intersected by membranous septa. From the arrangement of the bone in the larger mass, there seemed little doubt that it had been deposited in a similar tubercle. It consisted principally of phosphate of lime.

An oval pessary was found in the vagina, and from its appearance, and the intelligence obtained from the lady's daughter, it must have been left there for a great number of years. It had been entirely forgotten by the lady herself, and by her family.

She was the mother of three children, and at the time of her death the youngest of these was thirty-five years of age.

*No. 35, Mary-street, Hampstead-road.*

